# Alcohol Use Disorder and Traumatic Brain Injury

**DOI:** 10.35946/arcr.v39.2.07

**Published:** 2018

**Authors:** Zachary M. Weil, John D. Corrigan, Kate Karelina

**Affiliations:** Zachary M.Weil, Ph.D., is an assistant professor in the Center for Brain and Spinal Cord Repair and Group in Behavioral Neuroendocrinology, Department of Neuroscience, Ohio State University Wexner Medical Center, Columbus, Ohio. John D. Corrigan, Ph.D., is a professor in the Department of Physical Medicine and Rehabilitation, Ohio State University Wexner Medical Center, Columbus, Ohio. Kate Karelina, Ph.D., is a research scientist in the Center for Brain and Spinal Cord Repair and Group in Behavioral Neuroendocrinology, Department of Neuroscience, Ohio State University Wexner Medical Center, Columbus, Ohio

**Keywords:** alcohol and other drug use (AODU) development, AODU initiation, brain, injury, trauma

## Abstract

Alcohol use and traumatic brain injury (TBI) are inextricably and bidirectionally linked. Alcohol intoxication is one of the strongest predictors of TBI, and a substantial proportion of TBIs occur in intoxicated individuals. An inverse relationship is also emerging, such that TBI can serve as a risk factor for, or modulate the course of, alcohol use disorder (AUD). Critically, alcohol use after TBI is a key predictor of rehabilitation outcomes, prognosis, and additional head injuries. This review provides a general overview of the bidirectional relationship between TBI and AUD and a discussion of potential neuropsychological and neurobiological mechanisms that might underlie the relationship.

## Overview of Traumatic Brain Injury

Traumatic brain injury (TBI) is characterized by neurological dysfunction caused by a bump, blow, or penetrating injury to the brain. The duration and severity of dysfunction may range from “mild” TBI (concussion), which may involve a brief period of loss of consciousness and a transient neurological impairment with rapid recovery, to “severe” TBI, involving an extended period of loss of consciousness and permanent brain damage.^1^ The extent of neurological damage is determined by an evolving pathophysiology over the hours and days following the injury, during which time brain swelling, increased intracranial pressure, and reduced cerebral blood flow contribute to the development of cognitive and functional deficits.^2^ Further, the injuries can be divided into those that cause focal or penetrating damage to local brain regions versus those that result in more diffuse damage.^3^ Consequently, TBI is a highly heterogeneous injury state resulting in a patient population with markedly different injuries, comorbidities, and predicted outcomes.

Public understanding of TBI is currently undergoing a shift due, in part, to recent events that have focused public and media attention on the issue.^4^,^5^ Although these recent events, which include the emerging understanding of the role of TBI in later neurodegeneration and the recognition of the high incidence of TBI among amateur and public athletes, as well as military personnel, represent tragedies with real human cost, they have also helped focus public attention on an ongoing public health crisis.

Annually, about 2.8 million civilians in the United States receive medical treatment for TBI, but the true incidence of TBI is actually far higher, as many TBI patients are never seen by health care providers^6^,^7^ (although rates of emergency department visits are rising, likely due to increasing public awareness of the seriousness of TBI).^8^ Even among those patients seen by medical personnel, the lack of definitive diagnostic tools, or even consensus on a definition, means that a substantial proportion of TBIs go undiagnosed.^9^ Additionally, TBI was declared the signature injury among military personnel involved in the protracted conflicts in Iraq and Afghanistan (Operations Enduring Freedom, Iraqi Freedom, and New Dawn).^10^ During the first 12 years of these conflicts, nearly 250,000 service members were diagnosed with TBI,^11^ although the difficulties associated with reporting, identifying, and diagnosing head injuries indicate that this number likely is underestimated.

What is becoming clear, is that even relatively mild TBI can have far-reaching consequences that last well beyond the initial symptoms.^12^ The long-term sequelae of TBI can include psychiatric and neurological dysfunction, as well as a whole host of nonneurological diseases. Additionally, survivors of TBI can suffer from cognitive issues and are more likely to be unemployed, socially isolated, and incarcerated.^13^,^14^ Thus, the total cost, comprising health care dollars, loss of productivity, and quality of life, associated with TBI in the United States is substantial, with estimates of lifetime cost (in 2009 dollars) ranging from more than $75 billion to more than $200 billion.^15^

## Alcohol Use Disorder Before TBI

TBI has long been closely associated with acute alcohol intoxication. Most studies estimate that between 30% and 50% of patients treated for TBI were intoxicated at the time of injury, with even greater intoxication estimates for patients injured in motor vehicle accidents and assaults.^16^ Binge drinking is a major risk factor for trauma, particularly brain trauma.^17^ Individuals who consume more than five drinks in a sitting are more than three times as likely to suffer a trauma.^18^ One illustrative example involves cyclists. Individuals who cycle while intoxicated are more likely to fall, and, among cyclists who fall, being intoxicated greatly increases the probability of TBI.^19^ The lifetime incidence of TBI is approximately four times higher among individuals who drink, relative to those who do not.^20^

Not surprisingly, given the powerful relationship between alcohol intoxication and brain injuries, the overall rate of alcohol use disorder (AUD) is very high among patients who incur TBI, with estimates ranging from one-third to half of all patients meeting diagnostic criteria for AUD.^21^ More than half the patients admitted for rehabilitation following TBI meet the diagnostic criteria for AUD^22^ or are considered at risk for problem drinking because of self-reported binge drinking or Short Michigan Alcoholism Screening Test (SMAST) scores.^21^ Thus, the population of persons with TBI disproportionately consists of individuals who drink alcohol and those who meet AUD diagnostic criteria or are at risk for developing AUD.

Given that alcohol intoxication is a major risk factor for the incidence of TBI, a substantial population exists from which researchers can study the effects of blood alcohol concentration at time of injury on survival and on functional outcomes. There is controversial literature (beyond the scope of the current review) suggesting that better long-term outcomes are associated with patients who had low to moderate levels of alcohol in their blood at the time of their injuries, when compared with patients who had no alcohol in their blood,^23^,^24^ although not all studies have reached that conclusion.^25^ What is much clearer, however, is that drinking *after* TBI represents a major impediment to successful outcomes in several critical domains.^16^,^26^

## Patterns of Drinking After TBI

Alcohol use falls off immediately after TBI, and this reduction appears to be due to three factors.^21^ First, many patients are advised to abstain from alcohol in the early postinjury period to reduce the likelihood of post-traumatic seizures.^27^ Second, many patients with TBI have limited access to alcohol because they are hospitalized, living with family, or admitted to an inpatient rehabilitation facility, or because they have impairments in cognition or mobility.^21^ Finally, many patients, especially those whose injuries occurred secondary to intoxication, choose to use this early period to stop drinking. Indeed, involvement in car crashes increases the likelihood that patients will enter AUD treatment.^28^ Some patients stop drinking permanently, but a large subset (25%, by some estimates) resumes drinking after injury, and consumption levels can rise to (or above) preinjury levels by 1 to 2 years after injury.^29^ The strongest predictor of postinjury AUD is drinking before injury. Patients who scored high on the SMAST before TBI were more than 10 times likely to exhibit problem drinking after injury.^22^

There exists some controversy in the literature as to whether TBI can act as an independent risk factor for the development of AUD in adult patients who did not previously meet the diagnostic criteria for AUD.^30^,^31^ Epidemiological studies have generally concluded that TBI does not induce new cases of AUD, but some patients return to drinking after TBI (approximately 25%, by some estimates),^21^,^30^ and this has significant negative consequences (see **Consequences of Drinking After TBI** in this article). Still, there is reason to suspect that TBI can increase the likelihood of AUD. For instance, in one study, approximately 20% of patients who were abstainers or “light” drinkers before injury exhibited high-volume drinking after injury.^32^ Similarly, among military personnel, several studies have reported that service men and women who experienced combat-related TBI were more likely than uninjured individuals to binge drink.^33^ Additionally, among patients with a primary diagnosis of substance use disorder (defined as misuse of alcohol or drugs), a lifetime history of TBI is remarkably common. In one study of individuals seeking treatment for substance abuse in New York, more than 50% had a history of TBI, and nearly half had experienced more than one TBI.^34^

Still, any potential causal relationship between adult TBI and AUD has been difficult to establish for several reasons (although causality may exist). First, the TBI population disproportionately consists of people who exhibit AUD, potentially masking any relationship. Second, patients who have AUD after TBI appear more likely to be lost to follow-up in epidemiological and outcome studies.^35^ Third, patients who have the most severe injuries, the subset of people with TBI who, theoretically, are most likely to develop AUD, are also the group most likely to have no access to alcohol because of disability or institutionalization.^36^ Fourth, it is becoming increasingly clear that a large subset of patients treated for TBI also had previous TBI, and, as described in this article, injury during early development is a powerful risk factor for AUD.^37^ Fifth, the populations most at risk for TBI, including adolescent and young adult males, risk-takers, and enlisted military personnel, are also at elevated risk for AUD.^38^

The relationship between TBI and AUD is much clearer in individuals who were injured as children. Incurring TBI during childhood increases the likelihood of later development of AUD. This relationship is easier to discern because the effects of injury on the developing nervous system can be profound,^39^ and because this population is less affected by many of the confounders already discussed. Younger patients, presumably, are less likely to be experienced with alcohol or meet the diagnostic criteria for AUD.

For instance, results from the Christchurch birth cohort studies indicated that children who experienced mild TBI with hospitalization before age 5 were 3.6 times more likely to meet the *Diagnostic and Statistical Manual of Mental Disorders (Third Edition–Revised)* criteria for alcohol dependence during adolescence, when compared with those who had no similar injury.^40^ A 10-year, nationwide, longitudinal cohort study in Taiwan indicated that there was a more than sixfold increase in the rate of alcohol abuse (as defined by the *International Classification of Diseases, Ninth Revision: Clinical Modification*) among patients with a history of TBI, when compared with uninjured control patients.^41^ Among Canadian high school students, the odds ratio for binge drinking in the previous year (at the time of the study) was between two-and fourfold higher in students who had a history of TBI (defined as loss of consciousness or an overnight hospitalization), when compared with uninjured students.^42^ Moreover, in a study of patients admitted for inpatient rehabilitation following TBI, approximately 20% of the population had experienced previous TBI, many sustained before age 16.^37^ Among the patients in this study, those with a history of childhood brain injury had twice the rate of problem alcohol use as those without previous TBI. (Problem alcohol use was defined as more than 14 drinks per week for males and 7 for females, or any incidence of binge drinking that included 5 or more drinks in a night.)

Also, TBI appears to act indirectly by limiting protective factors and increasing risk factors for incurring a subsequent TBI.^43^ For instance, individuals with a history of TBI early in life are less likely to participate in extracurricular activities, finish school, marry, and be employed, and they are more likely to engage in risky behavior and experience long-term alienation from family and peer groups, all of which serve as risk modifiers for alcohol misuse.^37^,^44^,^45^ TBI, particularly when it occurs in young patients, can modify the risks for development of AUD, and, among individuals who have AUD, there is a high incidence of prior TBI.

## Comorbidity Among TBI, PTSD, and AUD

**Figure 1:**
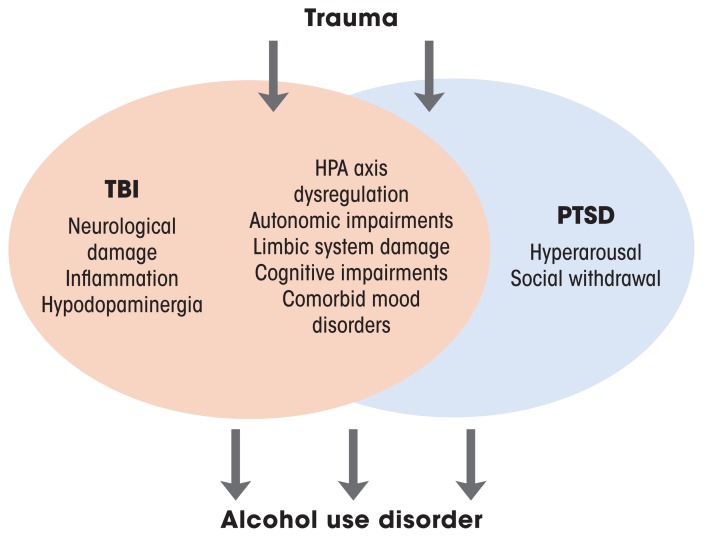
Overlapping neurobehavioral links among TBI, PTSD, and alcohol use disorder. TBI and PTSD share trauma as a precipitating event. They are also linked by dysregulation of stress response systems, cognitive impairments, and affective symptoms, which, together, can increase the likelihood of alcohol misuse. *Note:* HPA, hypothalamic pituitary adrenal; PTSD, post-traumatic stress disorder; TBI, traumatic brain injury.

TBI is closely linked to post-traumatic stress disorder (PTSD), but not only because both conditions have trauma as a precipitating factor (see Figure 1). Among combat veterans who had physical trauma excluding the brain, 16% developed PTSD symptoms, whereas 44% of combat veterans with a history of TBI developed symptoms of PTSD.^46^ Similar patterns have been observed among civilians.^47^ Remarkably, this relationship exists even among individuals who experienced post-traumatic amnesia that prevented them from remembering the trauma.^48^ The potential physiological links between the two conditions remain under investigation, but they may involve dysregulation of the hypothalamic pituitary adrenal axis, impairments in autonomic physiology, and damage to frontal and limbic structures that impair physiological regulation and the ability to manage traumatic memories.^49^,^50^

Critically, TBI, PTSD, and AUD are commonly comorbid, which is unsurprising given that intoxication elevates risk of TBI, and that generally high rates of alcohol misuse occur among patients who have TBI.^21^ The relationships among these conditions are an area of active investigation. Numerous studies have investigated relationships between two of the conditions, and far fewer have investigated all three.^51^ There are clearly relationships between and among all these conditions, but there are a number of overlapping characteristics of individuals with PTSD and TBI that can make drinking more likely.^52^ For instance, the hyperarousal to stressful events that is central to PTSD pathology is unpleasant and can increase social withdrawal, thus exacerbating ongoing negative affect.^52^ TBI can make it more difficult for patients to manage these symptoms, increasing the likelihood that they will drink alcohol. Moreover, the cognitive impairments combined with decreased frustration tolerance that are central to both TBI and PTSD can increase the likelihood that daily difficulties will lead to drinking. Because some of the relationship between TBI and AUD is likely mediated by PTSD, it has been difficult to disentangle the contribution of TBI and PTSD to the development of AUD, given their similar etiology and symptomatology. Further work is required to uncover the physiological substrates that link these conditions.

## Consequences of Drinking After TBI

Multiple epidemiological studies have reported that a subset of people with TBI eventually drinks at or above preinjury levels.^20^,^22^,^31^,^32^ This propensity to resume consuming alcohol at preinjury levels is of critical importance, because alcohol use after injury is deleterious in a number of different domains and is predictive of negative outcomes over the long term.^16^

A distinction has to be drawn between AUD and alcohol use in the absence of problem drinking. People who have brain injuries likely suffer negative consequences from patterns of drinking that would not produce significant harm in uninjured individuals. For instance, drinking can promote development of post-traumatic seizures directly and by interfering with the efficacy of prescribed antiseizure medications.^53^ Critically, alcohol affects peripheral tissues, including in the liver and kidneys, and impairs wound healing, which can have outsized effects on patients recovering from trauma. Also, cognitive consequences of drinking appear to be magnified by prior TBI. For instance, patients with TBI who drank at “heavy social” levels (with a mean Alcohol Use Disorders Identification Test score of 16.9) exhibited impaired event-related potentials and greater cognitive deficits, when compared with patients who abstained.^54^

Finally, both drinking and a history of TBI are powerful risk factors for suffering subsequent head injuries.^55^ Moreover, suffering TBI while intoxicated more than triples the likelihood of suffering a future TBI.^56^ Repeated TBIs produce more severe long-term damage and permanent disability than a single injury.^55^ Patients with TBI often report reduced tolerance to alcohol,^57^ and they can also have balance problems associated with their injuries, meaning that intoxication, even at relatively low blood ethanol concentrations, can increase the risk of injury.

Patients with AUD who continue (or restart) drinking after TBI have significantly poorer long-term outcomes than patients who do not.^58^ A chronic high level of drinking can be proinflammatory and deleterious to brain health and thus has the potential to impair functional recovery and further damage vulnerable and already impaired neural structures.^59^ Many of the brain regions commonly injured in TBI, including the frontal and medial temporal regions, are also key sites of inflammatory reactions in people who have been drinking alcohol for a long time. Patients with TBI who were previously diagnosed with AUD and relapsed had smaller frontal gray matter volumes within the first year after injury than patients who did not relapse.^60^ Finally, in a retrospective study of patients who had TBI, individuals who met the criteria for substance use disorder (including alcohol) at the time of their injuries were four times more likely to die from suicide than patients who did not meet the criteria.^61^

Some of the negative consequences of drinking after TBI may be related to treatment compliance. Patients with AUD are less compliant with TBI rehabilitation and have poorer rehabilitation outcomes than patients who do not have AUD.^16^ Patients with AUD are also more likely to have lower levels of life satisfaction.^62^ Alcohol misuse also impairs reintegration into the workforce after injury. Among people who have TBI, alcohol misuse is the most commonly cited reason for termination from a vocational placement program.^63^ Also, patients with TBI and AUD are more likely than patients with TBI who do not have AUD to meet the diagnostic criteria for mood disorders and less likely to return to work.^60^

Because of the many deleterious consequences associated with drinking alcohol after TBI, treating AUD in people with TBI has the potential to markedly improve outcomes and reduce the likelihood of devastating repeated injuries.

## Treatment of Co-Occurring TBI and AUD

There are special considerations for treating co-occurring AUD and TBI. As already mentioned, people who have TBI may be disproportionately vulnerable to negative consequences of alcohol misuse. However, there are unique challenges and opportunities for treatment of AUD among people with TBI. After their injuries, many patients with TBI significantly reduce the amount of alcohol they drink.^21^,^30^ Although a substantial subset (approximately 25%) of these individuals eventually returns to (or surpasses) preinjury drinking levels, this initial period of abstinence has been characterized as a “window of opportunity” for screening and intervention. There is limited, but generally positive, evidence that brief interventional strategies and cognitive-behavioral therapies can be effective in this population.^52^

Although screening and monitoring for AUD are key steps in the management of TBI, many patients, particularly those who do not receive specialized or follow-up care, are not assessed for AUD risk. Moreover, patients with TBI represent a special challenge for treatment of AUD. TBI is a heterogeneous condition, but there are certain brain regions that are more likely to be damaged because of their anatomical location. These regions include the key areas for cognitive control and executive function in the frontal and anterior temporal regions. Thus, it is extremely common after moderate to severe TBI to suffer from cognitive deficits, impaired emotional regulation, and difficulty focusing attention. Therefore, AUD treatment protocols must be tailored to address the specific challenges of this population.

Additionally, people with TBI have high rates of neuropsychiatric comorbidities, including depression, anxiety, and PTSD, all of which can promote alcohol misuse and complicate AUD treatment.^60^ Treatment for comorbid psychiatric disorders, particularly addiction, is more challenging in patients with a history of TBI, but the existing evidence indicates that treatments targeting both PTSD and comorbid alcohol dependence produced greater reductions in symptoms for both disorders than treatments for either condition alone.^64^

Moreover, the efficacy of drugs (e.g., disulfiram and naltrexone) approved specifically for treatment of AUD has been minimally investigated in the TBI population.^65^ These drugs are not contraindicated for people who have TBI, but medication for this population tends to require careful titration and close monitoring of responses. Also, the elevated risks of substance misuse should be considered when using medication to manage TBI symptoms in this patient population.

The pharmacological treatments for management of TBI fall into two general classifications.^66^ In the acute phase after injury, a small number of compounds are administered to manage symptoms and to (attempt to) reduce damage from the initial injury. In the later phases, several psychoactive compounds (e.g., cholinesterase inhibitors, stimulants, and amantadine) are prescribed to modulate cognitive symptoms, fatigue, and insomnia.^66^–^68^ Although little direct evidence indicates that these compounds can increase the likelihood of developing AUD, it is imperative to consider how their potential and efficacy are influenced by alcohol if they are to have appropriate clinical effects.

## Mechanisms Linking AUD to TBI

There are a number of potential mechanisms that link TBI to AUD across both cognitive and psychosocial domains. Further, there is mounting evidence that central inflammatory signaling can interact with deficits in neural reward systems, which may indicate that people with TBI are more vulnerable to developing AUD.

### Cognitive and psychosocial links

The incentive motivation theory of drinking predicts that individuals drink alcohol to either enhance positive affect (i.e., directly improve mood or facilitate socialization) or reduce negative affect (i.e., alleviate depression or anxiety).^69^ The decision to drink or not drink alcohol, as predicted by this theory, is based on weighing the perceived benefits against the potential costs, which may include legal and occupational issues, hangovers, monetary costs, and social pressures. However, people with TBI often have difficulty weighing the future costs of their actions. For instance, laboratory-based neuropsychological tests demonstrate that people who have frontal lobe injuries consistently have deficits in decision-making, as assessed by their performance in delay discounting and gambling tasks that require judgment about future consequences of immediate actions.^70^,^71^ This pattern of cognitive deficits is superficially similar to what occurs in patients with AUD, and these cognitive deficits are worse in patients with TBI who meet the diagnostic criteria for AUD.^72^ Thus, despite future negative consequences, people with TBI may be less likely than those without TBI to decide to not drink.

### Neurobiological substrates

Neurobiological links between TBI and AUD remain unspecified, although a potential link has received increased attention in recent years, and new animal models have been developed.^73^,^74^ Injury to the brain often results in affective, cognitive, and psychosocial impairments that can promote alcohol misuse. Moreover, the underlying neurobiological roots of these impairments may also render the brain more vulnerable to developing alcohol dependence.

To investigate the potential relationship between TBI during development and future alcohol use, we developed an animal model in which we administered a mild TBI to mice during juvenile development and allowed the animals to grow into adults.^75^ Animals that experienced TBI as juveniles exhibited markedly greater alcohol self-administration as adults, when compared to noninjured animals. The difference in alcohol self-administration between the two groups of animals was independent of changes in sensory function. Also, for the mice that had TBI, the difference was associated with enhanced reward responses to intraperitoneal alcohol. Thus, the injury during juvenile development altered the rewarding properties of alcohol. Moreover, we could block the enhanced drinking behavior that followed TBI by housing the animals in enriched environments, which served as a proxy for sustained cognitive and physical rehabilitation. We have begun to use this model to investigate the neurobiological substrates of alterations in alcohol-related circuitry.

For instance, as already discussed in this article, TBIs are remarkably heterogeneous in etiology, location, and severity, but they do possess some common features.^3^ Specifically, virtually all TBI produces acute neuroinflammatory response and persistent alterations in neuroimmune physiology.^76^ This is important because alcohol and central inflammatory responses are bidirectionally linked. High doses of alcohol produce a characteristic inflammatory response in the brain, including activation of microglia and upregulation of proinflammatory signaling molecules.^59^ Further, this inflammatory response to alcohol is exacerbated in animals with a history of TBI. We recently showed that mice that experienced TBI during juvenile development exhibited exaggerated inflammatory responses, cognitive deficits, and neural degeneration following binge-like alcohol administration in adulthood.^77^ Moreover, inflammatory responses in the brain drive alcohol-drinking behavior in animals, and blocking or reducing neuroinflammatory signaling can attenuate alcohol self-administration.^78^–^80^ Thus, we postulate that TBI establishes a state of constant escalation in which it directly induces an inflammatory response and also enhances the neuroinflammatory response to subsequent exposure to alcohol.^73^ Future studies need to address whether inhibiting TBI-induced inflammatory responses can also prevent increases in drinking alcohol.

TBI also may produce a state of hypodopaminergia. In clinical populations, imaging data and the widespread use of dopaminergic agents (e.g., methyl-phenidate and amantadine) for the treatment of TBI-related cognitive issues provide indirect evidence of the hypodopaminergia.^14^ Whether the effectiveness of dopaminergic agents in patients with TBI reflects a true dysregulation of mesocorticolimbic dopamine, or if higher dopaminergic tone is beneficial for cognitive function in survivors of TBI, remains unspecified. However, in animals, TBI produces a biphasic alteration in dopamine signaling characterized by an initial upregulation of dopaminergic synthesis pathways and dopamine release, followed by prolonged suppression.

Neuroinflammatory responses have significant antidopaminergic effects,^81^ and blunted dopaminergic release is a major risk factor for the development of AUD.^82^ In our juvenile TBI model, injured mice exhibited markedly attenuated dopaminergic signaling in adulthood and altered patterns of neuronal activation in dopaminergic cells.^83^ There are many unanswered questions, but injury during periadolescent development in mice seems to persistently alter the development of the dopaminergic system and the response to alcohol in this key reward system. Clearly, there are many other mechanisms beyond neuroinflammation and hypodopaminergia that could underlie greater vulnerability to AUD in people with TBI, and this review is limited in scope.

## Future Research Needs

There are many unanswered questions regarding the relationship between TBI and AUD. Most pertinently, we need to determine if TBI exacerbates AUD or increases vulnerability to the development of AUD. We also need to ascertain how underlying neural mechanisms affect TBI and AUD. In particular, what are the roles of chronic neuroinflammatory signaling, impairments in reward processing, and cognitive issues in mediating susceptibility to AUD? We know that many people with TBI meet the diagnostic criteria for AUD and continue to drink alcohol after their injuries. Further, we know this pattern of behavior is associated with varied, but serious, negative consequences. Thus, future research needs to address the best ways to screen and treat people with TBI to minimize the harm associated with drinking alcohol after injury.
